# Raman spectroscopy-based prediction of ofloxacin concentration in solution using a novel loss function and an improved GA-CNN model

**DOI:** 10.1186/s12859-023-05542-3

**Published:** 2023-10-30

**Authors:** Chenyu Ma, Yuanbo Shi, Yueyang Huang, Gongwei Dai

**Affiliations:** 1 School of Information and Control Engineering, Liaoning Petrochemical University, Fushun, 113001 China; 2 School of Artificial Intelligence and Software, Liaoning Petrochemical University, Fushun, 113001 China

**Keywords:** Raman spectroscopy, Ofloxacin, Kernel-Huber loss function, Genetic algorithm-convolutional neural network, Concentration prediction

## Abstract

**Background:**

A Raman spectroscopy method can quickly and accurately measure the concentration of ofloxacin in solution. This method has the advantages of accuracy and rapidity over traditional detection methods. However, the manual analysis methods for the collected Raman spectral data often ignore the nonlinear characteristics of the data and cannot accurately predict the concentration of the target sample.

**Methods:**

To address this drawback, this paper proposes a novel kernel-Huber loss function that combines the Huber loss function with the Gaussian kernel function. This function is used with an improved genetic algorithm-convolutional neural network (GA-CNN) to model and predict the Raman spectral data of different concentrations of ofloxacin in solution. In addition, the paper introduces recurrent neural networks (RNN), long short-term memory (LSTM), bidirectional long short-term memory (BiLSTM) and gated recurrent units (GRU) models to conduct multiple experiments and use root mean square error (RMSE) and residual predictive deviation (RPD) as evaluation metrics.

**Results:**

The proposed method achieved an $R^2$ of 0.9989 on the test set data and improved by 3% over the traditional CNN. Multiple experiments were also conducted using RNN, LSTM, BiLSTM, and GRU models and evaluated their performance using RMSE, RPD, and other metrics. The results showed that the proposed method consistently outperformed these models.

**Conclusions:**

This paper demonstrates the effectiveness of the proposed method for predicting the concentration of ofloxacin in solution based on Raman spectral data, in addition to discussing the advantages and limitations of the proposed method, and the study proposes a solution to the problem of deep learning methods for Raman spectral concentration prediction.

## Introduction

Ofloxacin is a synthetic quinolone [1] with excellent antibacterial properties. However, its excessive use has resulted in increased resistance of bacteria and other microorganisms [2], reducing its efficacy against bacterial infections. The abuse of ofloxacin in farming has become frequent in recent years. Humans who consume food with excessive antimicrobial drugs may develop symptoms such as diarrhea and headache, which pose a threat to human health [3]. Several countries have introduced regulations and standards to control the use and residues of ofloxacin. For example, China banned the use of antibacterial drugs such as ofloxacin in farming [4] and restricted the use of key antibacterial drugs for zoonotic diseases. Therefore, accurate and rapid detection methods for monitoring ofloxacin levels in food are important. Regression analysis model is a common prediction method [5], and this paper aims to use an improved CNN regression analysis model to predict the ofloxacin level and evaluate its performance.

Raman spectroscopy has many applications in fields such as biomedicine and food safety because of its easy operation [6], fast detection, and high accuracy [7]. Traditional methods for processing Raman spectra, such as partial least squares regression (PLSR) and principal component regression (PCR), assume linearity and ignore the nonlinear characteristics of spectral data [8]. These methods are also susceptible to noise and background interference, which can lead to poor prediction accuracy. Moreover, factors such as human errors and instrument errors during Raman spectral data acquisition can affect the analysis of nonlinear relationships between spectral concentrations [9]. Recently, with the rapid development of artificial intelligence technology, machine learning (ML) methods have been used to process Raman spectroscopy data due to their excellent predictive performance [10]. Liu et al. [11] predicted the concentration of benzo(a) pyrene in peanut oil using support vector machine (SVM) algorithm, and the results outperformed traditional methods such as PLSR. Lin et al. [12] developed a slurry concentration prediction model using an artificial neural network (ANN) approach, and the results showed that ANN had better nonlinear predictive ability than PLSR. ML methods can solve some nonlinear problems, but they have limitations when dealing with more complex nonlinear relationships in real data [13]. Deep learning [14], especially the wide application of CNN and graph convolutional networks (GCN) [15, 16], aims to overcome the limitations of traditional ML methods. In comparison to traditional ML methods, deep learning has stronger generalization ability and better performance in handling nonlinear problems [17]. CNN, a classical algorithm of deep learning, is widely used in many fields because of their superior feature extraction [18]. While Wu et al. [19] employed CNN to predict honey concentration for authentication, it exhibited reduced robustness with limited data. Pian et al. [20] enhanced accuracy and robustness by applying the residual connectivity technique to CNN for quantitative blood glucose analysis and prediction. Chen et al. [21] proposed a regression analysis method with loss function combined with kernel function. The method used Gaussian kernel function to compensate for the weak nonlinear characteristics of the mean square error (MSE) loss function.

Raman spectra acquisition generates noise and outliers, which the Huber loss function can address by combining squared and absolute errors [22]. To further improve the regression model’s robustness, we propose a new Gaussian kernel Huber loss function that more accurately measures the deep learning error’s nonlinearity and handles noise or outliers better [23]. Combining intelligent algorithms with neural network algorithms has become a main research direction in recent years to address the difficulty of parameter control in neural network building [24]. Therefore, we propose a concentration prediction model of CNN with an improved adaptive step size genetic algorithm and Gaussian kernel Huber loss function, aiming to achieve fast and accurate prediction of the ofloxacin solution concentration.

## Algorithm model

### Kernel-Huber loss function

Traditional linear regression methods can predict common regression problems effectively. However, they have limitations when faced with nonlinear problems. For example, the instrumental and manual biases in the Raman spectroscopy acquisition process may cause noise [25], which makes the nonlinear features of the actual data unfitted and affects the final results. CNN can automatically extract and learn features from the data and extract more advanced features through layer-by-layer learning, thus enabling nonlinear modeling. This study uses an improved CNN algorithm and proposes a new loss function (Kernel-Huber Loss) based on the Huber loss function and Gaussian kernel function. The new loss function has better robustness in the regression task and can balance the fitting and generalization ability of the model, thus improving the model prediction accuracy.

#### Traditional Huber loss function

The Huber loss function is a regression loss function that reduces the outlier sensitivity and avoids their excessive influence on the model [26]. The basic principle of the traditional Huber loss function as shown in Eq. (1).

1 $$\begin{aligned} {L_\delta }(y,f(x)) = \left\{ {\begin{array}{*{20}{l}} {\frac{1}{2}{{(y - f(x))}^2}}&{}\quad {{\text {if }}|y - f(x)| \le \delta }\\ {\delta |y - f(x)| - \frac{1}{2}{\delta ^2}}&{}\quad {{\text {otherwise}}} \end{array}} \right. \end{aligned}$$

where *y* is the real data, *f*(*x*) is the predicted data, and $\delta$ is the truncation tolerance that measures the difference between *y* and *f*(*x*). The squared error measures the error when $|y - f(x)| \le \delta$, and the linear error similar to mean absolute error(MAE) measures the error otherwise. However, the traditional Huber loss function may not handle outliers or noisy data well when the data distribution is complex or noisy.

#### Kernel-Huber loss function

The human error of the collected Raman spectral data and the molecular interaction can disrupt the linear relationship between Raman peak intensity and concentration, particularly when concentrations are too high [27]. An effective method for considering the nonlinear characteristics of spectral concentration prediction is to map the input data to a high-dimensional space and perform vector inner product operations using a kernel function to mitigate the curse of dimensionality. Specifically, the original data is mapped to the Hilbert Space [28] for calculation, with $\phi$ as the feature mapping function, and the inner product calculation formula in Eq. (2):

2 $$\begin{aligned} K(y,f(x)) = \langle \phi (y),\phi (f(x))\rangle \end{aligned}$$

Gaussian kernel function and polynomial kernel function are widely used in ML. Gaussian kernel function is more flexible than polynomial kernel function. It can handle nonlinear separable data and does not change the relative position between data points [29], which makes it excellent in many ML tasks such as classification, regression, etc. The Gaussian kernel function is shown in Eq. (3).

3 $$\begin{aligned} K(y,f(x)) = \exp \left( { - \frac{{\left( y - f(x)\right) {^2}}}{{2{\sigma ^2}}}} \right) \end{aligned}$$

The Huber loss function combined with the Gaussian kernel function can be written as Eq. (4).

4 $$\begin{aligned} {L_{}}({y_{}},f(x)) = \frac{{\sum \nolimits _{i,j} K ({y_{(i)}},f{{(x)}_{(j)}}){L_\delta }({y_{(i)}},f{{(x)}_{(i)}})}}{{\sum \nolimits _{i,j} K ({y_{(i)}},f{{(x)}_{(j)}})}} \end{aligned}$$

where ${y_{(i)}}$ represents the data value in the original input space, $f(x)_{(i)}$ stands for the predicted value for the ith input data point, and $f(x)_{(j)}$ denotes the predicted value after mapping to the new feature space.

### Residual connected CNN

#### 1D depthwise separable convolution

**Fig. 1:**
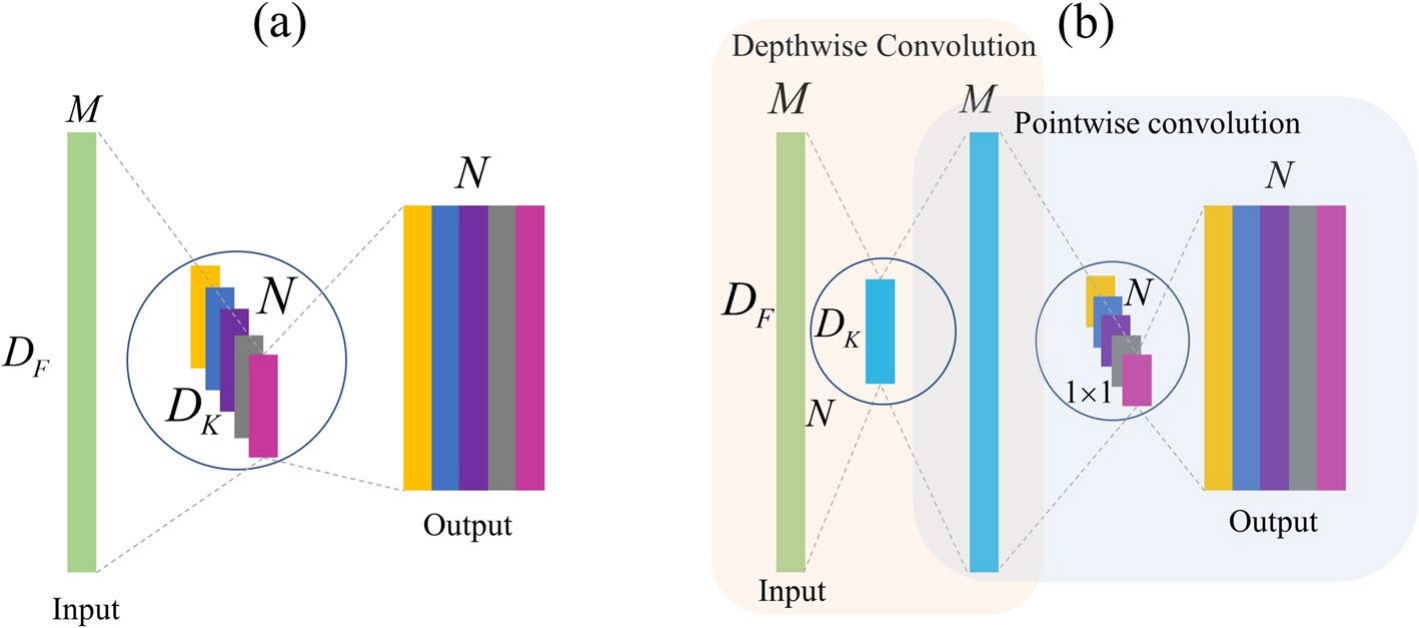
**a** Standard 1D convolution schematic and **b** 1D Depthwise schematic

To solve the problem of gradient vanishing and exploding in deep neural networks, the residual network is used to optimize the neural network based on the improved CNN. The one-dimensional depthwise separable convolution decomposes the ordinary convolution process into two independent convolution processes, which can reduce the parameter number and the overfitting phenomenon by decomposition [30]. In short, 1D depthwise separable convolution decomposes the ordinary convolution process into two steps: depthwise convolution and pointwise convolution. First, depthwise convolution is applied to each input channel of Raman spectral input data, and then pointwise convolution is applied to the results of different channels. This is illustrated in Fig. 1.

The ratio of the computational effort of 1D Depthwise Separable Convolution to ordinary convolution is shown in Eq. (5).

5 $$\begin{aligned} \frac{{{D_{k}} \times M \times {D_{F}} + M \times N \times {D_{F}}}}{{{D_{k}} \times M \times N \times {D_{F}}}} = \frac{1}{N} + \frac{1}{{{D_{k}}}} \end{aligned}$$

where ${D_{k}}$ represents the convolution kernel size, ${D_F}$ represents the input data length, when the number of convolution kernels is taken as *K* and the number of input feature data channels is *M*, the number of parameters can be reduced by about $\frac{1}{{{D_{k}}}}$.

#### Design of residual structures

**Fig. 2:**
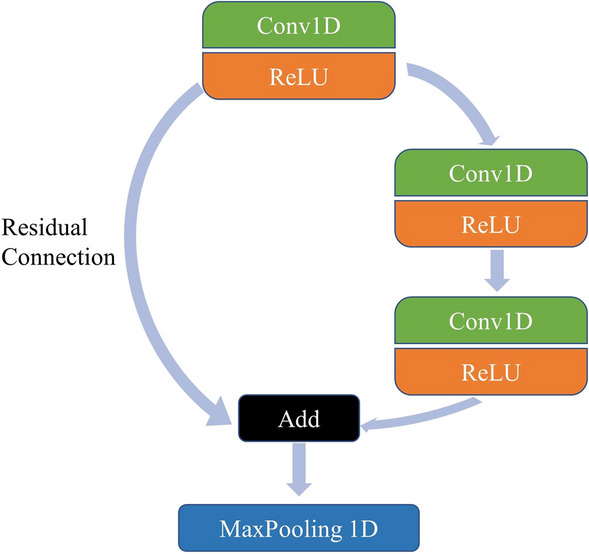
The schematic diagram of residual connection

The residual connection in neural networks allows the deeper extraction of Raman spectral feature information in the network [31], and the residuals can superimpose the nonlinear variation of the input data, which reduces the gradient vanishing in deep neural networks. The residual connection’s basic structure is illustrated in Fig. 2.

### Adaptive GA-CNN

**Fig. 3:**
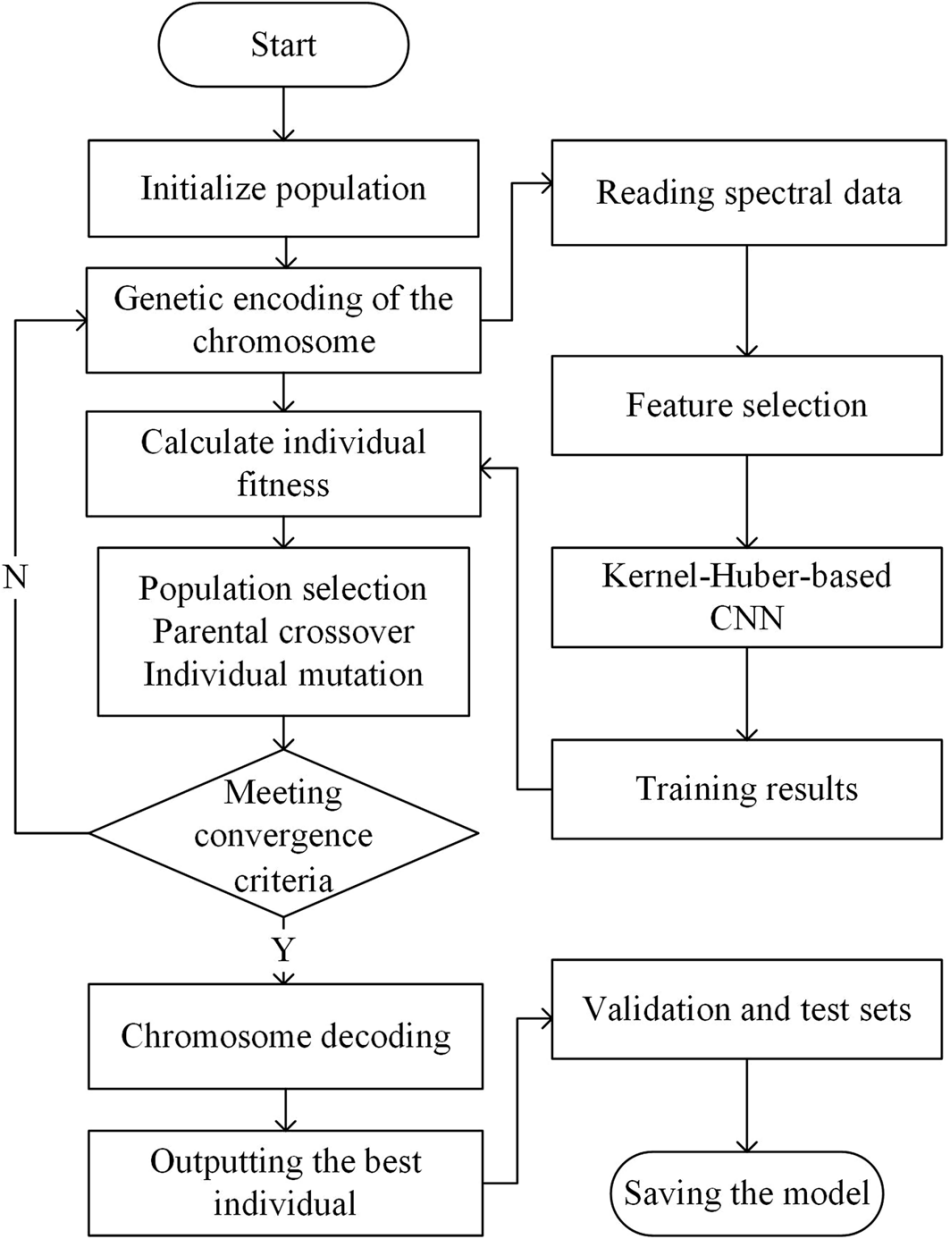
The structure of the prediction model for the concentration of of ofloxacin

The GA-CNN combined model applies a genetic algorithm to optimize an improved CNN. Since Raman spectral data exhibit nonlinear characteristics, this model leverages the selection and crossover operations of the genetic algorithm to find the global optimal solution for the weights of the CNN [32], thus achieving better nonlinear modeling. The model aims to build a Raman spectral concentration prediction model by using an optimized CNN. The genetic algorithm creates initial populations based on the initial weight matrix of the CNN to be optimized, and each population contains information on all values in the CNN. The GA-CNN model uses the error between the actual value and the predicted value as a fitness function. It iterates through the processes of population selection, crossover and mutation, and eventually obtains the optimal weights of the individual with the minimum error as the initial weights for GA-CNN. Moreover, it introduces an adaptive improvement for the crossover and mutation probabilities of the conventional GA to achieve faster convergence [33], as shown in Eq. (6), and the detailed procedure of the GA-CNN model is shown in Fig. 3.

6 $$\begin{aligned} {P_c} = \left\{ {\begin{array}{*{20}{l}} {{P_{c\min }} + \frac{{\left( {{P_{c\max }} - {P_{c\min }}} \right) \left( {1 - \frac{i}{G}} \right) }}{{1 + {e^{A\frac{{2\left( {f - {f_{avg}}} \right) }}{{{f_{\max }} - {f_{avg}}}}}}}}}&{}{\mathrm{{if }}\,\,f \ge {f_{avg}}}\\ {{P_{c\max }}}&{}{\mathrm{{ }}f \le {f_{avg}}} \end{array}} \right. \nonumber \\ {P_m} = \left\{ {\begin{array}{*{20}{l}} {{P_{m\min }} + \frac{{\left( {{P_{m\max }} - {P_{m\min }}} \right) \left( {1 - \frac{i}{G}} \right) }}{{1 + {e^{A\frac{{2\left( {f - {f_{avg}}} \right) }}{{{f_{\max }} - {f_{avg}}}}}}}}}&{}{\mathrm{{if }}\,\,f \ge {f_{avg}}}\\ {{P_{m\max }}}&{}{\mathrm{{ }}f \le {f_{avg}}} \end{array}} \right. \end{aligned}$$

The equation shows the values of ${P_c}$ and ${P_m}$, which are the crossover and mutation probabilities. The upper and lower limits of ${P_c}$ are ${P_{c\max }}$ and ${P_{c\min }}$, and the upper and lower limits of ${P_m}$ are ${P_{m\max }}$ and ${P_{m\min }}$. The equation also includes the individual fitness *f*, the average fitness ${f_{avg}}$ and the maximum fitness ${f_{max}}$. Finally *i* represents the current iteration number and *G* represents the maximum number of iterations for the genetic algorithm.

## Data acquisition and processing

### Experimental reagents

Sodium citrate (C$_{6}$H$_5$Na$_{3}$O$_{7}$, Belgium ACROS company), ofloxacin (content $\ge$ 99%). The hexane, chloroform and ethyl acetate used in the experiment were all analytically pure, carboxymethyl cellulose (CMC, Shanghai Tixi Chemical Trading Co, Ltd.) and diatomite. Ultra-pure water was used in all the experiments.

### Preparation of SERS-enhanced substrates

The glassware for preparing silver nanoparticles (AgNPs) was first immersed in aqua regia (HNO$_{3}$/HCl, 1:3, v/v) for 20 min and then rinsed with ultrapure water. AgNPs were prepared by the sodium citrate reduction method [34]. 1 mL of 0.1 m/L AgNO$_{3}$ aqueous solution was heated under reflux with stirring. Then, 4.0 mL of 1% sodium citrate was quickly added to the refluxing solution and the reflux continued for 30 min before cooling to room temperature. The prepared spherical AgNPs had a diameter range of 50–60 nm.

### Experimental method

A gradient solution of ofloxacin (loxacin dissolved in hydrochloric acid) ranging from 100 to 1 ppm was prepared and spotted on a diatomaceous earth plate 1.5 cm from the bottom of the thin layer chromatography plate. The sample was separated by placing the plate vertically in the mobile phase at room temperature and then the retention factor was calculated by tracking the position of the analyte using a UV lamp (254 nm) with iodine colorimetry. Then, 3 $\upmu$L of 30-fold concentrated silver nanoparticles were deposited on the analyte spots. Plots were made using Origin 2017. For rapid detection in the field, a portable Raman spectrometer (BWS465-iRman; B &W-Tek, USA) with a 785 nm excitation laser was used with a laser power of 30 mW and an integration time of 2 s. The Raman spectral data were saved as a csv file.

## Results and discussion

### Experimental setup

**Fig. 4:**
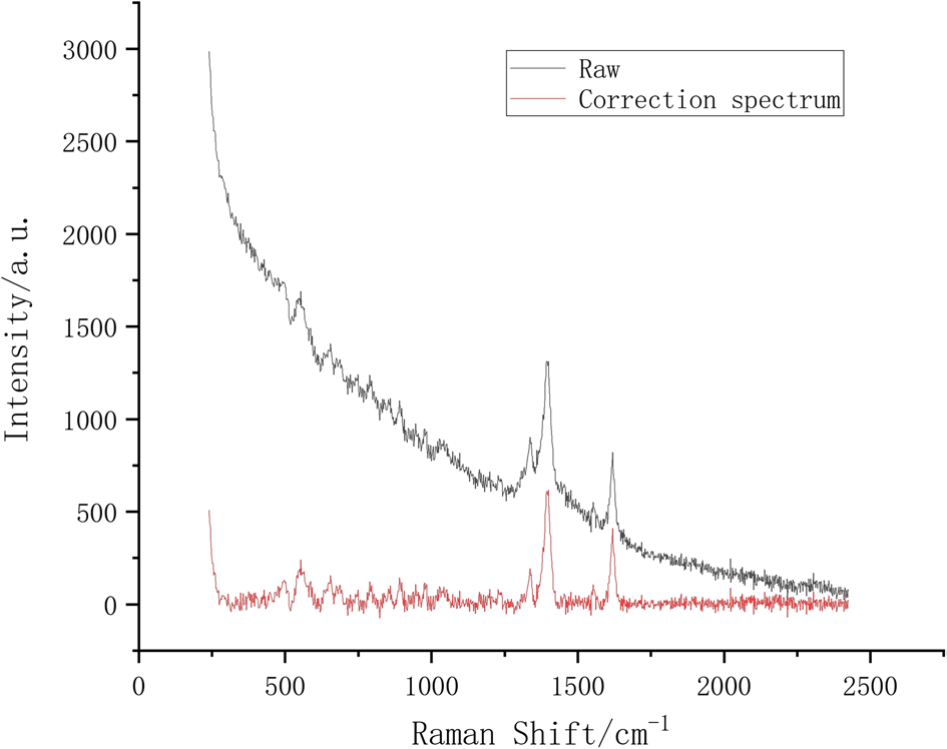
Comparison chart of preprocessing algorithm results

To effectively extract the spectral feature peak information and remove the fluorescence interference, baseline correction is required for the Raman spectral data. The adaptive iteratively reweighted penalized least squares (airPLS) [35] method is chosen for the baseline correction. Figure 4 shows the spectra of 100 ppm after airPLS baseline correction, ranging from 239 to 2400 cm$^{-1}$. In this range, the distribution of spectral features and characteristic peaks is relatively dense, whereas characteristic peaks at other wavelength positions are not apparent at low concentrations. The intensity of the characteristic peaks at (519–617 cm$^{-1}$) and (1292–1713 cm$^{-1}$) is significantly higher than the rest of the positions, which provides an important theoretical basis for accurate Raman spectral concentration classification. Therefore, the characteristic peaks in these ranges are selected for analysis and used as characteristic variables.

### Evaluation indicators for quantitative analysis models

The MSE, RMSE, mean absolute percentage error (MAPE) of the training and test sets can measure the accuracy, performance and robustness of the proposed Raman spectral concentration analysis model. The higher the RPD indicator value, the more accurate the model is [36], and the higher the median absolute error (MedAE) indicator value, the less robust the model is. Eqs. (7), (8), (9), (10), (11), (12) show the specific calculation formulas.

7 $$\begin{aligned}{} & {} R^2 = 1 - \frac{\sum _{i=1}^n (y_i - {\hat{y}}_i)^2}{\sum _{i=1}^n (y_i - {\bar{y}})^2} \end{aligned}$$

8 $$\begin{aligned}{} & {} {\text{MedAE}}(y,{\hat{y}}) = {\text{median}}(|y_1 - {\hat{y}}_1|, \ldots ,|y_n - {\hat{y}}_n|) \end{aligned}$$

9 $$\begin{aligned}{} & {} {\text{MAPE}} = \frac{100\%}{n} \sum _{i=1}^n \left| \frac{y_i - {{\hat{y}}_i}}{y_i} \right| \end{aligned}$$

10 $$\begin{aligned}{} & {} {\text{RMSE}} = \sqrt{\frac{1}{n} \sum _{i=1}^n (y_i - {\hat{y}}_i)^2} \end{aligned}$$

11 $$\begin{aligned}{} & {} {\text{RMSEC}} = \sqrt{\frac{1}{m} \sum _{j=1}^m (y_j - {{\hat{y}}_j})^2} \end{aligned}$$

12 $$\begin{aligned}{} & {} RPD = \frac{1}{\sqrt{1 - R^2}} \end{aligned}$$

where *m* and *n* represent the number of samples in the training and test sets, respectively. ${y_i}$ and ${{{\hat{y}}}_i}$ represent the actual and predicted concentrations of the *i*th sample in the test set, respectively; ${y_j}$ and ${{{\hat{y}}}_j}$ represent the actual and predicted concentrations of the *j*th sample in the training set, respectively.

**Table 1:** Model comparison

Models	$\textrm{R}^{2}$	MedAE	MAPE	RMSE	RPD	RMSEC
OURS	0.9989	0.7650	2.8404	1.0207	29.8872	1.4909
CNN	0.9689	3.4168	27.3789	5.3790	5.6717	5.9278
GRU	0.9855	2.8889	16.1136	3.6696	8.3138	5.7840
LSTM	0.9562	4.7007	28.3233	6.3840	4.7788	6.0532
RNN	0.9466	7.1858	30.7423	7.0519	4.3262	4.8422
BiLSTM	0.9753	3.5844	15.3264	4.7926	6.3656	6.2974

In this paper, five neural network architectures were compared: CNN, GRU, LSTM, RNN and BiLSTM for predicting the concentration of ofloxacin from its Raman spectrum. 51 Raman spectral data samples of ofloxacin were collected, of which 40 were used for training and 11 for testing. Models were developed for each of these datasets under the same preprocessing conditions (least squares for baseline correction), and Table 1 summarizes the results. As Table 1 shows, the model proposed in this paper outperforms other concentration prediction methods in all aspects. It has the highest ${R^2}$ and the lowest MedAE, MAPE and RMSE values for the test sets. In addition, its RPD values are significantly higher than those of other algorithms. The comprehensive analysis indicates that the Kernel-Huber-based CNN has high accuracy and robustness. This means that the model can effectively fit the training sets data and generalize well to the test sets data, avoiding overfitting or underfitting problems.

### Performance of Kernel-Huber-based CNN compared to other algorithms

**Fig. 5:**
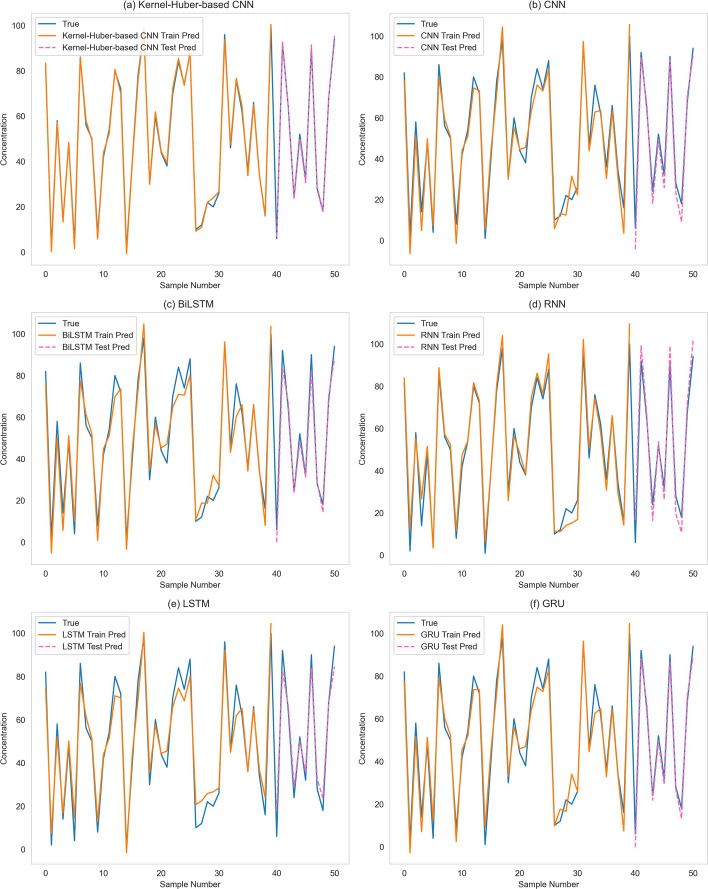
Comparison between real value and measured value

**Fig. 6:**
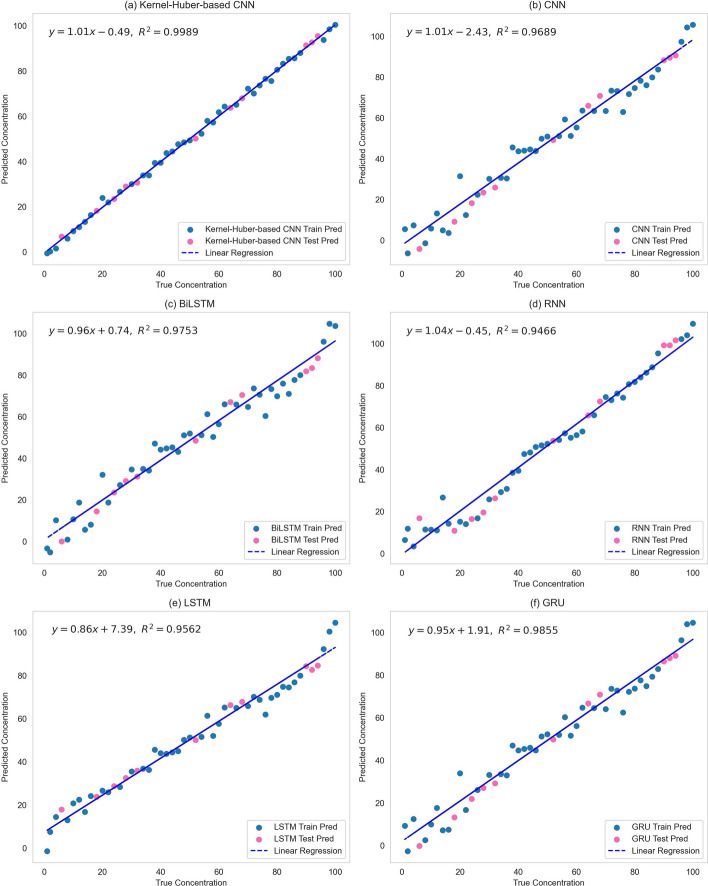
Relationship between predicted ofloxacin concentration and actual ofloxacin concentration

To more intuitively represent the performance of the Kernel-Huber-based CNN in concentration prediction, Fig. 5 shows the prediction results of various models. At the same time, to verify the actual performance effects and differences of the six models, scatter plots of the predicted values versus the actual values of each model on the training and test datasets are plotted separately for comparison, and linear regression equations are fitted as Fig. 6 shows.

Figure 5 shows that under the same amount of data as the training data condition of the model, the accuracy of the Kernel-Huber-based CNN proposed in this paper is significantly higher than the other five models. The kernel function allows patterns that would otherwise be linearly indistinguishable in lower dimensions to be linearly differentiable in higher dimensions by mapping the data into a higher dimensional space. It helps to solve complex prediction problems. In Fig. 5, the Kernel-Huber-based CNN shows a slight deviation in predicting 20ppm concentration, while the other five algorithms deviate at multiple concentrations, as shown in Fig. 5b–f. Figure 6 also shows that the Kernel-Huber-based CNN has the best prediction performance. It achieves the highest ${R^2}$ value in all datasets, and the predicted and actual values are closely distributed on both sides of the linear regression line. This fully demonstrates that the Kernel-Huber-based CNN has a better performance in extracting Raman spectral features and is more reliable in dealing with the Raman spectral concentration prediction problem.

### Adaptive GA algorithm

**Fig. 7:**
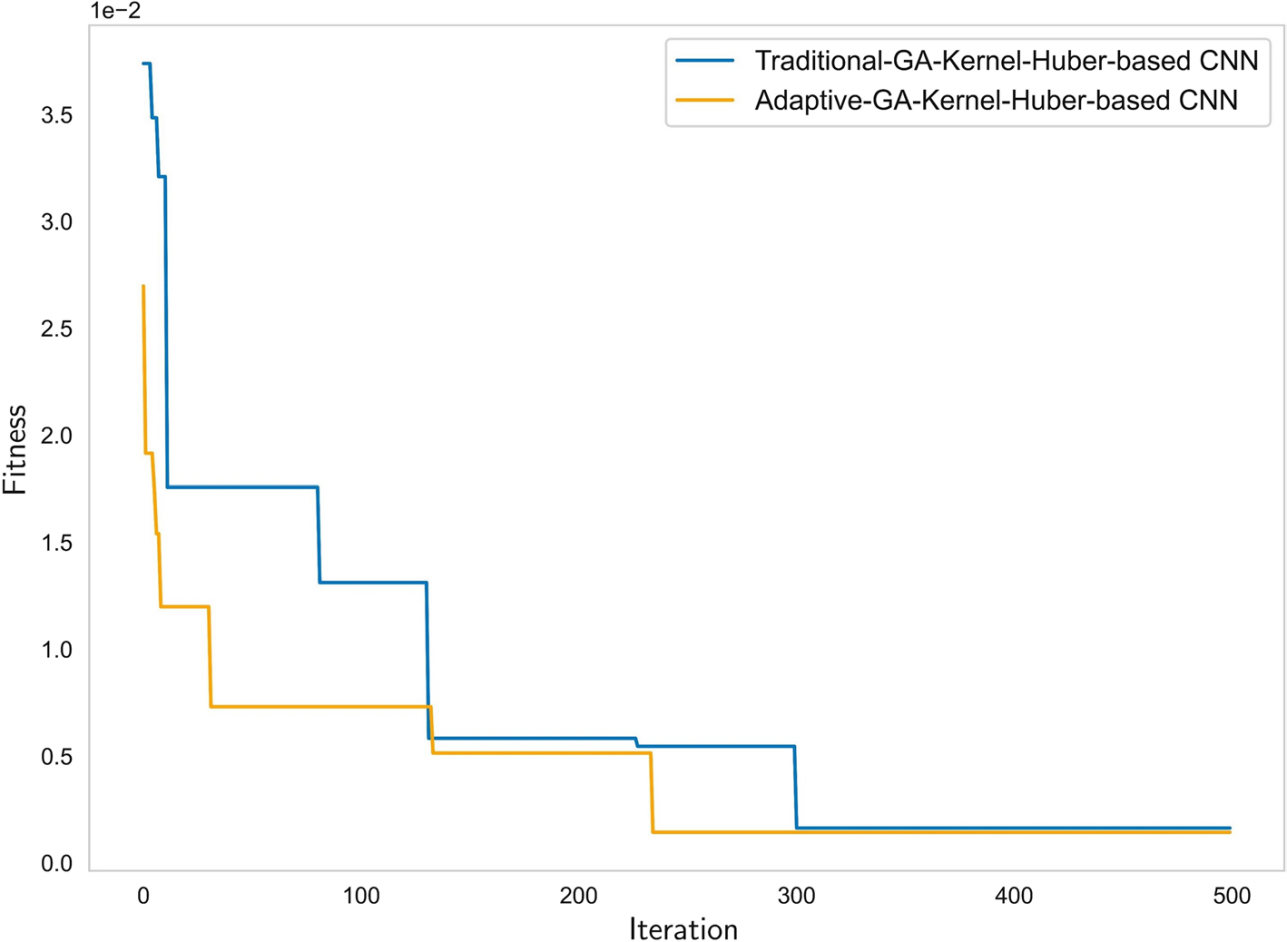
Comparison between traditional-GA and adaptive-GA

To verify the effectiveness of the concentration prediction method based on the improved adaptive genetic algorithm proposed in this paper and to compare its performance with the traditional genetic algorithm. As shown in Fig. 7, we chose the difference between the actual and predicted concentrations of the test set as the fitness function. The convergence curves of the two algorithms show that their fitness function values are similar because they both use Kernel-Huber-based CNN, but the improved adaptive genetic algorithm converges faster. This is because it adapts the crossover and mutation probabilities according to the fitness values and iteration numbers, which enhances the exploration ability of the model. Too high or too low probabilities will reduce the performance of the algorithm.

## Conclusion

This paper introduces an adaptive genetic algorithm with improved loss function model for predicting the concentration of ofloxacin. By employing a Gaussian kernel function, the model improves the robustness of prediction and extracts the non-linear patterns in Raman spectral data more effectively. The model also improves the crossover and mutation probability adjustment strategies, and adds the catastrophe and mutation operations, which enhance the convergence speed and global optimization finding ability. Although this study has potential applications, the computational complexity of the algorithm may increase with the increase of data size. Future research could focus on further enhancing the model’s parameter adaptive tuning strategy to alleviate parameter tuning challenges and enhance the model’s practical applicability. This study provides directions and insights for future research in the field of drug concentration prediction.

## Data Availability

All the code used to prepare the data and fit the models is available at https://github.com/Mchyu/Novel-Loss-Function-and-an-Improved-GA-CNN.
